# An evaluation of emergency guidelines issued by the World Health Organization in response to four infectious disease outbreaks

**DOI:** 10.1371/journal.pone.0198125

**Published:** 2018-05-30

**Authors:** Susan L. Norris, Veronica Ivey Sawin, Mauricio Ferri, Laura Raques Sastre, Teegwendé V. Porgo

**Affiliations:** 1 WHO Guidelines Review Committee, World Health Organization, Geneva, Switzerland; 2 National School of Public Health, Madrid, Spain; 3 Department of Social and Preventative Medicine, Laval University, Quebec City, Canada; Katholieke Universiteit Leuven Rega Institute for Medical Research, BELGIUM

## Abstract

**Background:**

The production of high-quality guidelines in response to public health emergencies poses challenges for the World Health Organization (WHO). The urgent need for guidance and the paucity of structured scientific data on emerging diseases hinder the formulation of evidence-informed recommendations using standard methods and procedures.

**Objectives:**

In the context of the response to recent public health emergencies, this project aimed to describe the information products produced by WHO and assess the quality and trustworthiness of a subset of these products classified as guidelines.

**Methods:**

We selected four recent infectious disease emergencies: outbreaks of avian influenza A—H1N1 virus (2009) and H7N9 virus (2013), Middle East respiratory syndrome coronavirus (MERS-CoV) (2013), and Ebola virus disease (EVD) (2014 to 2016). We analyzed the development and publication processes and evaluated the quality of emergency guidelines using AGREE-II.

**Results:**

We included 175 information products of which 87 were guidelines. These products demonstrated variable adherence to WHO publication requirements including the listing of external contributors, management of declarations of interest, and entry into WHO’s public database of publications. For guidelines, the methods for development were incompletely reported; WHO’s quality assurance process was rarely used; systematic or other evidence reviews were infrequently referenced; external peer review was not performed; and they scored poorly with AGREE II, particularly for rigour of development and editorial independence.

**Conclusions:**

Our study suggests that WHO guidelines produced in the context of a public health emergency can be improved upon, helping to assure the trustworthiness and utility of WHO information products in future emergencies.

## Introduction

The World Health Organization (WHO) shapes public health policy for United Nations Member States through the development of technical guidance. More than a decade ago, in response to harsh external criticism of its guidelines, WHO instituted the Guidelines Review Committee (GRC) with the mandate to develop rigorous methods and procedures ensuring that each WHO guideline is evidence-based and meets the highest international standards so that guidelines are credible, trustworthy and relevant to end-users [1]. WHO quality standards dictate that guidelines must address a critical public health problem, use transparent and explicit processes minimizing potential sources of bias such as conflicts of interest, include diverse perspectives in the guideline development group, reflect the current state of the evidence, and provide a clear link between the evidence and recommendations taking into consideration the balance of benefits and harms of interventions and other important considerations [2].

WHO’s scope of work includes producing guidelines and other information products in the context of public health emergency response such as natural and technological disasters, disease outbreaks, armed conflicts and other humanitarian crises. When relevant guidelines do not exist, either because the situation is new or because current guidelines are not applicable, it is extremely challenging to produce new guidelines. Significant uncertainty in the field leads to urgent need, imposing short development timelines; scientific data are scarce and the collection of new data is hindered; health systems may be poorly functioning or nonexistent; and resources (both money and expertise) for guideline development may be scant. Despite this challenging context, adherence to the principles and standards for high-quality, trustworthy and usable guidelines is essential such that end-users adopting and implementing recommendations in the field can optimally impact population health outcomes.

The best way to apply traditional quality standards for guidelines to the context of emergencies remains unclear. WHO does not currently have an established approach with defined processes and tools to develop emergency guidelines. Thus, guideline development groups at WHO and in other organizations must improvise and adapt standard methods on an *ad hoc* basis, potentially compromising the quality of the final product. To date there are no comprehensive evaluations of guidelines produced in the context of a public health emergency. The aim of this study is to describe the information products produced by WHO in recent emergencies and to assess the quality and trustworthiness of a subset of these products classified as guidelines. The overarching goal of this evaluation was to inform future processes and methods for producing these types of guidelines at WHO and by other organizations globally.

## Methods

### Inclusion criteria for information products

We examined information products issued by WHO for four recent public health emergencies: outbreaks of avian influenza A—H1N1 virus (2009), avian influenza A—H7N9 virus (2013), Middle East respiratory syndrome coronavirus (MERS-CoV) (2013) and Ebola virus disease (EVD) (2014 to 2016). There were other outbreaks, humanitarian emergencies, and natural disasters between 2009 and 2016 that were graded by the Global Emergency Management Team [3], however we selected these four emergencies as purposeful sample as they all focused on infectious diseases outbreaks, and involved a significant response by WHO including the production of a large number of information products. All occurred after the institution of the GRC so that a standard approach to develop guidelines in the non-emergency context was in place at WHO.

We included all information products published in any language that were specifically developed or adapted by WHO for the four selected emergencies. We excluded testimonials, media releases, situation reports and meeting reports. Two independent reviewers assessed each document against study inclusion criteria. As per WHO’s definition [2], we classified an information product as a guideline based on the content of that document (i.e., whether it contained a specific recommendation for the end-user), rather than by the process for development. Guidelines included products that contained recommendations and did not reference an underlying, source guideline. These covered a wide range of topics including clinical care [4], water and sanitation [5], and occupational health and safety [6], among many other topics. All other information products were classified as non-guidelines, which included, for example, publications derived from existing guidelines [7], assessments of preparedness in countries[8], and tools for assessment or response [9]. Non-guidelines are not expected to contain detailed methods and other types of information that is expected in a high-quality guideline.

### Identification of included information products

We identified eligible information products available on 24 April 2016, from lists of technical documents related to each emergency assembled by the WHO Department of Communicable Disease Surveillance and Response. We had difficulty identifying relevant documents and therefore searched multiple potential sources such as WHO's global digital library (Institutional Repository for Information Sharing (IRIS)), hand-searched WHO websites, consulted key WHO staff in technical units and reviewed WHO resource toolkits.

### Data extraction and analysis

Two independent reviewers extracted information product characteristics and publication format, and identified translations. For guidelines, we extracted data on standard WHO guideline development processes (e.g., based on a systematic review of the evidence, involved external experts in the formulation of recommendations) and had two independent reviewers appraise each guideline using the Appraisal of Guidelines for Research and Evaluation (AGREE) II instrument[10]. This instrument, the most widely-used guideline quality appraisal tool, is comprised of 23 items, six quality domains and a 7-point Likert-like response scale.

We report the prevalence of key characteristics for information products in each emergency. For continuous data, we report the mean and standard deviation (SD). We summarize AGREE II scores for each domain (scaled to a percentage of the maximum score) across guidelines using the median value and the interquartile range, as scores were not normally distributed and sample sizes were small for some emergencies. All statistical analyses were conducted using Stata 12.1 (StataCorp LP, College Station, Texas, USA).

## Results

We identified 175 information products of which 87 were classified as guidelines. Table 1 summarizes the characteristics of all information products. Table 2 outlines the methods and processes used in the development of guidelines specifically.

**Table 1:** Characteristics of information products produced by WHO in four recent public health emergencies.

	H1N1 (2009)		H7N9 (2013)		MERS-CoV (2013)		EVD (2014–16)	
	Guideline	Non-guideline	Guideline	Non-guideline	Guideline	Non-guideline	Guideline	Non-guideline
Number	15	39	5	9	16	7	52	32
Publication year	2009: 11 2010: 3 2011: 1	2009: 36 2010: 2 2011: 1	2013: 4 2014: 1 2015: 0	2013: 8 2014: 0 2015: 1	2013: 2 2014: 4 2015: 10	2013: 2 2014: 4 2015: 0	2014: 28 2015: 18 2016: 5	2014: 15 2015: 15 2016: 1
Translations available^a^	0 (0)	0 (0)	0 (0)	1 (11)	11 (73)	0 (0)	32 (65)	10 (32)
Downloadable format ^a^	15 (100)	37 (95)	5 (100)	9 (100)	15 (94)	6 (86)	49 (94)	31 (97)
Title contains: "guideline" or "guidance" "interim"	3 (20) 2 (13)	1 (3) 0 (0)	1 (20) 2 (40)	0 (0) 0 (0)	8 (50) 9 (56)	0 (0) 0 (0)	25 (48) 19 (37)	6 (19) 4 (13)
Number of pages, mean (range)	13 (2–39)	6 (1–72)	7 (2–13)	11 (1–51)	6 (1–22)	31 (4–94)	24 (1–102)	19 (2–49)
Involved external individuals	8 (53)	31 (79)	3 (60)	7 (78)	5 (31)	4 (57)	27 (52)	16 (50)
Provided publication date	13 (87)	27 (69)	5 (100)	8 (89)	13 (81)	6 (86)	51 (98)	31 (97)
Included the WHO logo ^a^	14 (93)	34 (92)	3 (60)	8 (89)	15 (100)	4 (67)	47 (96)	28 (90)
Provided contact details for the responsible officer or team	1 (7)	12 (31)	1 (20)	7 (78)	4 (25)	4 (57)	5 (10)	2 (6)
Located in IRIS ^a^	1 (7)	0 (0)	0 (0)	0 (0)	8 (53)	0 (0)	45 (92)	25 (81)

EVD, Ebola virus disease; IRIS, Institutional Repository of Information Sharing; MERS-CoV, Middle East Respiratory Syndrome Coronavirus; SD, standard deviation; WHO, World Health Organization. Data represent number (%) unless otherwise indicated.

^a^ Of information products for which a downloadable file was available (H1N1: 15 guidelines, 37 non-guidelines; H7N9: 5 guidelines; 9 non-guidelines; MERS-CoV: 15 guidelines, 6 non-guidelines; EVD: 49 guidelines, 31 non-guidelines).

**Table 2:** Guideline development processes in recent public health emergencies.

	H1N1 n = 15	H7N9 n = 5	MERS-CoV n = 16	EVD n = 52
References a systematic or rapid review	2 (13)	1 (20)	0 (0)	3 (6)
Contains citations to other documents	12 (80)	4 (80)	12 (75)	42 (81)
Lists external contributors	4 (27)	1 (20)	4 (25)	10 (19)
Reports collecting declarations of interest	3 (20)	1 (20)	1 (6)	4 (8)
Peer review conducted	2 (13)	0 (0)	6 (38)	5 (10)
States the date of expiration or planned update	8 (53)	0 (0)	3 (19)	4 (8)
States the source of funding	0 (0)	0 (0)	0 (0)	5 (10)
Approved by the WHO Guidelines Review Committee	1 (7)	0 (0)	0 (0)	2 (4)

EVD, Ebola virus disease; MERS-CoV, Middle East Respiratory Syndrome Coronavirus; n, number; SD, standard deviation

WHO, World Health Organization.

Data represent number (%) unless otherwise indicated.

### H1N1

**Fig 1:**
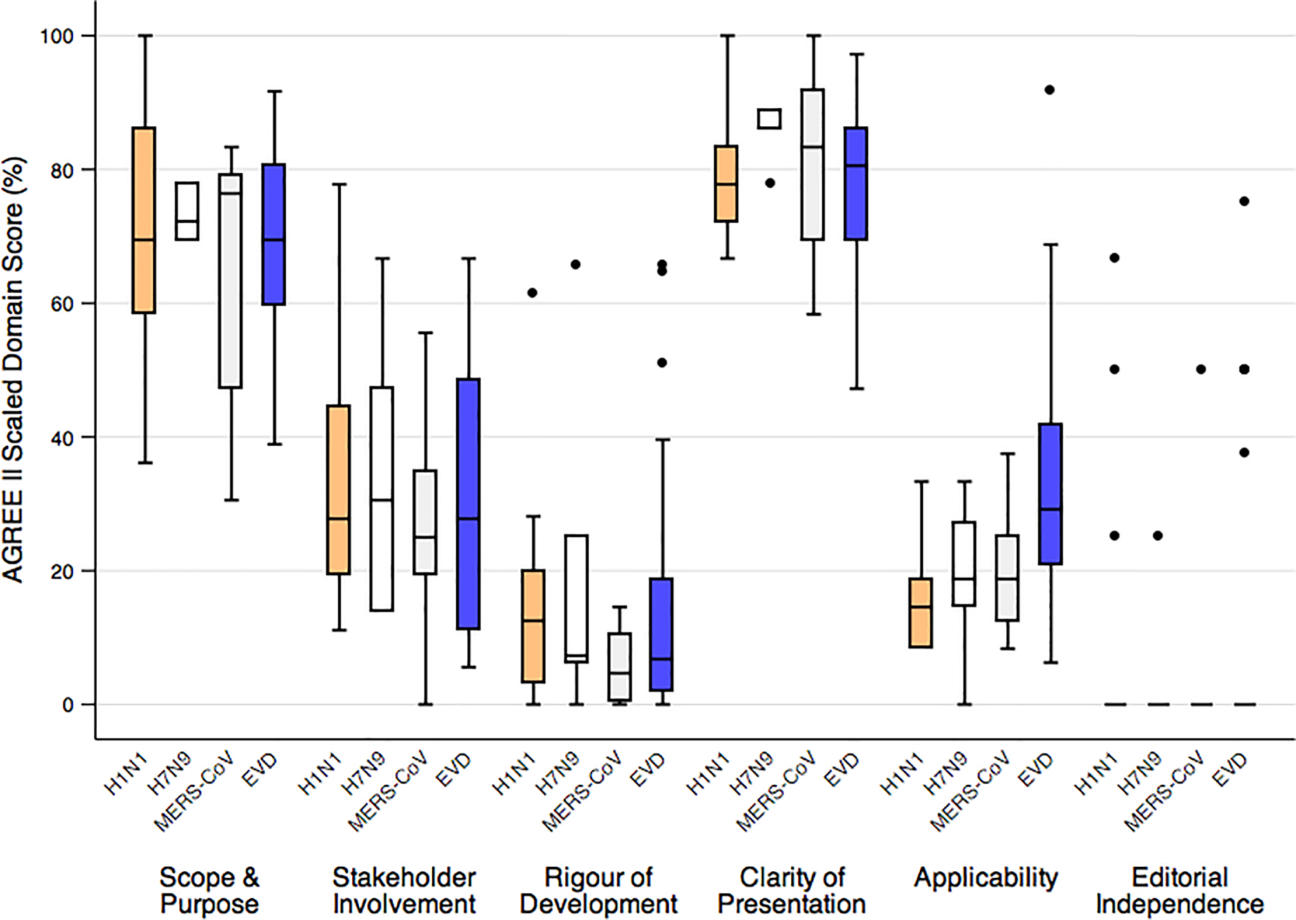
AGREE II scaled domain scores of guidelines developed for H1N1, H7N9, MERS-CoV and EVD. EVD, Ebola virus disease; MERS-CoV, Middle East Respiratory Syndrome Coronavirus. The AGREE II domains are displayed along the horizontal axis. The data points are the median, interquartile range, and minimum and maximum standardized scores based on the scores of the two reviewers for each domain and outbreak.

For the H1N1 emergency response, 54 information products met our inclusion criteria: 15 guidelines and 39 other information products [11]. Systematic reviews were cited in only two guidelines (13%) and four other guidelines (27%) referred to a literature search. External experts were involved in 53% of guidelines, but only four named these contributors of which three reported that declaration of interests (DOI) had been obtained. Peer review rarely occurred. Dates of expiration or planned update for the guidelines were provided in eight documents (53%). H1N1-related guidelines received the highest scores in the AGREE II domains of clarity of presentation (median 78%; interquartile range (IQR) 74%-83%) and scope and purpose (median 69%; IQR 63%-85%) (Fig 1). The lowest scores were assessed in editorial independence (median 0%; IQR 0%-0%), rigour of development (median 13%; IQR 4%-20%), and applicability (median 15%; IQR 9%-19%).

### H7N9

For H7N9, we identified 14 emergency information products, five emergency guidelines and nine other information products. All but one document was available only in English. One guideline referenced a systematic review; another cited evidence, but did not indicate whether the evidence review was systematic. Of the three guidelines that reported involving external experts, only one listed those individuals and provided their DOI. None of the guidelines provided a date of expiration or planned update, though one guideline indicated that recommendations would be updated as new information emerged. Based on the AGREE II instrument, H7N9 emergency guidelines scored well in clarity of presentation (median 89%; IQR 86%-89%) and scope and purpose (median 72%; IQR 69%-78%). The lowest-scoring AGREE II domains were rigor of development (median 7%; IQR 6%-25%), applicability (median 19%; IQR 15%-27%) and editorial independence (median 0%; IQR 0%-0%) (Fig 1).

### MERS-CoV

Twenty-three information products, including 16 emergency guidelines and seven other information products, met our inclusion criteria for MERS-CoV documents. Eleven guidelines (73%) were available in at least two languages (English and Arabic); we identified only an English version of the remaining information products. None of the MERS-CoV emergency guidelines referenced a systematic review. Although five guidelines (31%) reported that external experts contributed to the recommendations, only four of these listed the names of individual experts and only one reported that DOI had been collected. Three guidelines (19%) provided a specific date for the expiration or update of the guideline. The AGREE II domains that scored the highest across these MERS-CoV-related emergency guidelines were clarity of presentation (median 83%; IQR 72%-92%) and scope and purpose (median 76%; IQR 53%-78%). The lowest scoring domains were editorial independence (median 0%; IQR 0%-0%) and rigour of development (median 5%; IQR 1%-9%) (Fig 1).

### EVD

We included 84 EVD information products, including 52 emergency guidelines and 32 other information products. Overall, 42 (50%) information products were available in English and French, including 32 (62%) guidelines and 10 (31%) non-guidelines; the remainder were available only in English. Only two EVD guidelines reported or referenced a systematic review, although a third guideline reported that a rapid review of the evidence had been performed. An additional five guidelines (10%) referenced evidence or indicated that a literature search had been performed, but it was unclear if the review methods were systematic. Four guidelines (8%) reported that recommendations were based entirely on expert opinion. Most guidelines (81%) cited other publications, including other WHO information products. Four guidelines (8%) reported that DOI had been collected from external experts. Four guidelines provided a date for the expiration or update of the guideline; eight additional guidelines indicated that regular updates would be provided as new information became available. EVD guidelines scored the highest in the AGREE II domains of clarity of presentation (median 81%; IQR 69%-86%) and scope and purpose (median 69%; IQR 60%-81%). The lowest scores were assessed in editorial independence (median 0%; IQR 0%-0%) and rigour of development (median 7%; IQR 2%-18%) (Fig 1).

**Fig 2:**
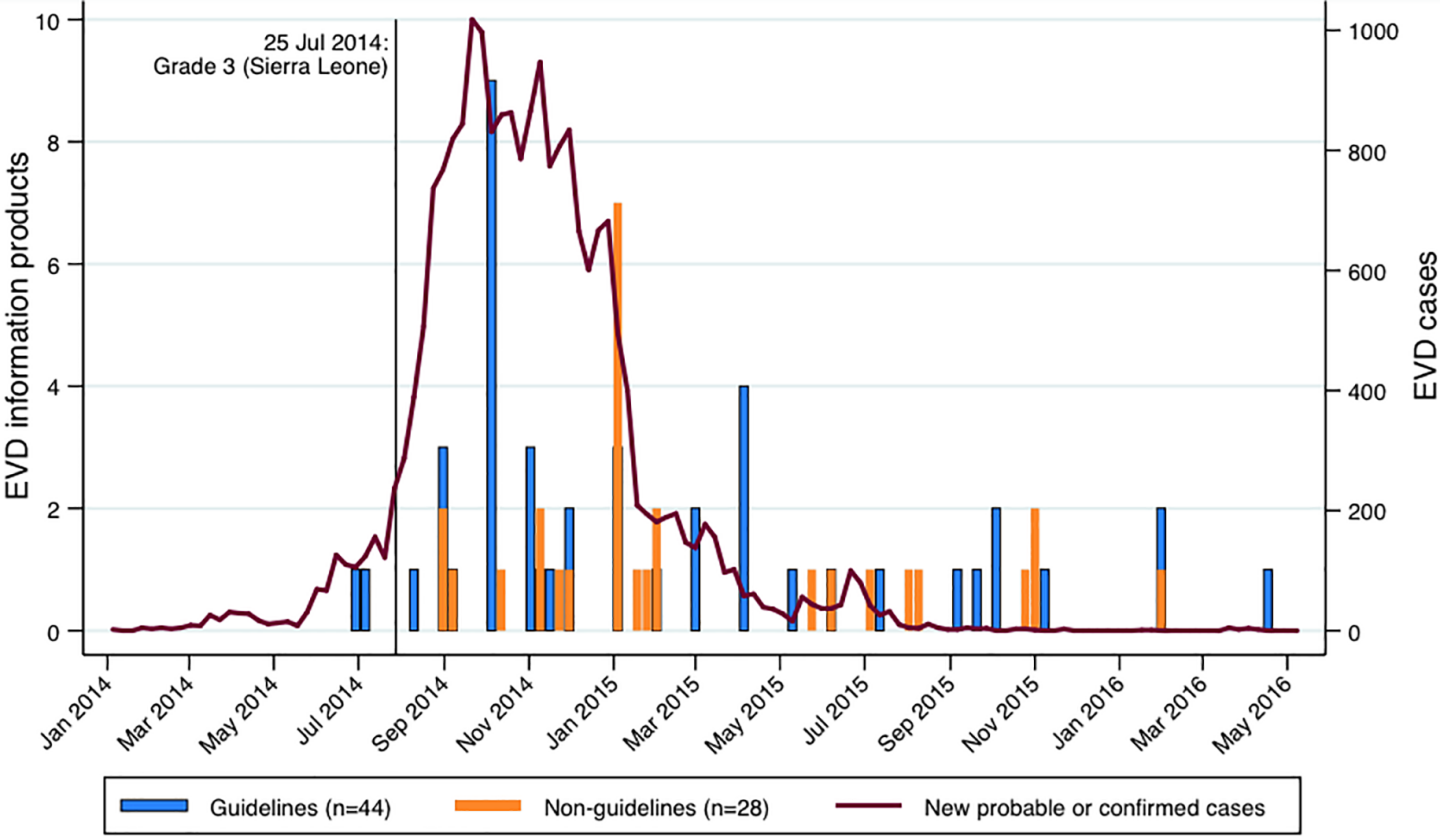
Ebola virus disease information products and timing of the epidemic. EVD, Ebola virus disease.

Approximately half (51%) were published in 2014 (Fig 2). Guidelines were published an average of 178 days (SD 200) following the initial emergency grading of EVD in Sierra Leone on 25 July 2014; two documents (4%) were published prior to the assignment of the emergency grade.

## Discussion

Information products issued by WHO during four recent infectious disease epidemics were difficult to find, were often published at long intervals after outbreaks were graded, and demonstrated variable adherence to WHO publication and reporting requirements. Few guidelines met international standards for the development and presentation of trustworthy and usable guidelines, as assessed with tools designed for standard (non-emergency) guidelines.

The principles for trustworthy guidelines were developed in the context of standard (non-emergency) clinical guidelines where there is a much longer time-period for development, end-users’ needs may be much clearer, there may be much more (high-quality) evidence, and the implementation setting and health systems may be much less fragile and chaotic [1]. Universally agreed-upon standards and methods for the optimal development of public health guidelines in the context of emergencies have not yet been established and thus our assessments using these standards must be interpreted with caution. Nonetheless, the principles of transparency, minimization of risk of bias in the formulation of recommendations, and the presentation of implementable, impactful recommendations inarguably apply to all types of guidelines and recommendations.

The difficulty we faced to identify and retrieve relevant documents suggests that end-users may have similar or greater difficulties during the emergency response. In addition, incomplete reporting of key information such as the responsible technical unit and publication date may prevent end-users from determining the relevance of a product to their specific context as the epidemic trajectory changes or new information emerges.

There was no consistent format of the information products that we examined, including within specific emergencies. Information products should vary in their content and appearance to meet the needs of the intended end-users, however WHO products should be readily identifiable with consistent appearance and branding, including the WHO logo. The labelling of information products varied across products of the same or similar types, even within emergencies. In particular, guidelines were referred to by a vast array of terms that did not appear to have a specific meaning. For example, “interim guidance” was relatively frequently used in EVD-related documents, appropriately suggesting a limited shelf-life for these documents, however specific plans for updating were rarely presented.

We noted incomplete implementation of WHO’s mandatory policy on conflicts of interest of external contributors and the reporting of funding sources for guidelines. This is an important finding as the interests of both author and funder are correlated with the conclusions of systematic reviews and the recommendations in clinical practice guidelines [12]. Although DOI does not in itself minimize the risk of bias associated with those interests, disclosure is widely regarded as the first and essential step in addressing competing interests and their potential effects on the conclusions of an evidence review or the recommendations in a guideline.

Despite the WHO GRC’s responsibilities, only three guidelines were approved by the quality assurance agency. The specific reasons were unclear, although the pressure to produce guidelines quickly could lead to *ad hoc* and variable processes and procedures. Nonetheless, quality assurance and control processes must still be used for all WHO information products.

Although incomplete reporting likely impeded our assessment, the rigour of development of the guidelines examined in our study appears to be low, as assessed by AGREE II and infrequent reference to systematic reviews. Compressed timelines in response to urgent needs necessitate abbreviated methods, however an explicit description of the approaches used to develop a guideline is always possible. The information and considerations used to inform each recommendation must be clearly described and when direct evidence is scarce, it is even more important to be transparent about the basis for recommendations. The applicability of indirect evidence must be carefully assessed and the rationale for its inclusion provided (for example, the use of data from related viruses or other infectious agents with similar vectors).

Draft guidelines rarely underwent peer review by individuals and organizations distinct from the primary contributors to the guideline. This is an important gap in the development process, as peer review is a standard step in guideline development [1,2], and has important benefits including stakeholder engagement, identification of errors and attention to key implementation issues. Peer review can be executed within days and comments can be quickly evaluated with careful planning and ready access to a cohort of experts and stakeholders.

A clear indication of plans for updating recommendations is a critical issue in the emergency context, as the evidence base and other considerations such as those related to implementation are continually evolving. All information products developed in response to an emergency, particularly those labelled “interim”, should have a “review by” date and sufficient resources must be available to evaluate and update information products as required.

High scores in the AGREE II domains of clarity of presentation and scope and purpose may be attributable to the operational focus of most emergency guidelines, which typically requires a concise, clear format. Other AGREE II domain scores suggest significant room for improvement. For example, guidelines scored inconsistently in the domain of applicability, which includes criteria such as descriptions of barriers and facilitators to implementation of the recommendations, and provision of or reference to other tools to support implementation.

Although this evaluation examines a number of important measures, there are many additional questions that were not addressed. We focused on emergency information products for infectious disease outbreaks and it is important to examine information products developed in response to other types of emergencies such as natural disasters and humanitarian crises caused by displacement of populations secondary to conflict or climate change. We did not evaluate dissemination, utilization, perceived usefulness by end-users, implementation and uptake of recommendations, or most importantly, the impact on health outcomes and well-being. Such impact evaluations are needed. Nor did we try to identify gaps in the guidance covered by the available information products.

## Limitations

Despite our efforts to compile comprehensive lists of information products developed by WHO in response to H1N1, H7N9, MERS-CoV and EVD, we may not have captured all relevant documents. We examined only the latest version available: some information products may have been updates which may have different characteristics than previous versions that were developed in shorter timelines or earlier in the emergency response. For practical reasons, we evaluated only information reported in the documents. We may have misclassified derivative products as guidelines if they failed to reference the underlying guideline.

Although widely used to assess the quality of reporting and conduct of guidelines, the AGREE II instrument has limitations [13], and it was developed for clinical guidelines and not for public health or emergency guidelines. Thus, the domain scores that we report should be interpreted with caution. The AGREE II tool is also limited in its scope. For example, the domain of applicability examines the inclusion of tools for implementation and discussion of barriers, cost implications, and monitoring of the guideline’s recommendations, but it does not address actual implementation or impact on health. In addition, we had only two assessors for the AGREE II appraisals; although acceptable according to AGREE II guidance, four reviewers would have enhanced the reliability of these assessments [10].

This study did not intend to compare the quality of guidelines for the four responses with each other or with standard WHO guidelines developed in stable settings. It is challenging to compare guidelines across the four epidemics as each event differed with respect to incidence, mortality rate, rate of change in the burden of disease, geographic spread, prior experience with the disease, and the availability of resources.

## Conclusion

WHO guidelines produced in the context of a public health emergency can be improved upon, helping to assure the trustworthiness and utility of WHO information products in future emergencies. The principles of transparency, minimization of the risk of bias, and reliance on research and other evidence over expert opinion, apply to guidelines developed in any setting or context. With careful self-evaluation, thoughtful reflection on lessons learned in these emergencies, and a firm commitment to continuous improvement, WHO will continue to provide timely and high-quality guidance in the context of public health emergencies.

## Supporting information

S1 Table Individual-level AGREE-II scores. (XLSX)
