# Correction: An evaluation of emergency guidelines issued by the World Health Organization in response to four infectious disease outbreaks

**DOI:** 10.1371/journal.pone.0202782

**Published:** 2018-08-16

**Authors:** Susan L. Norris, Veronica Ivey Sawin, Mauricio Ferri, Laura Reques Sastre, Teegwendé V. Porgo

The fourth author's name is spelled incorrectly. The correct name is: Laura Reques Sastre. The correct citation is: Norris SL, Sawin VI, Ferri M, Reques Sastre L, Porgo TV (2018) An evaluation of emergency guidelines issued by the World Health Organization in response to four infectious disease outbreaks. PLoS ONE 13(5): e0198125. https://doi.org/10.1371/journal.pone.0198125
